# Phenotypic heterogeneity and cooperation in the metastatic cascade

**DOI:** 10.18632/oncoscience.565

**Published:** 2022-09-22

**Authors:** Katherine M. Young, Cynthia A. Reinhart-King

**Keywords:** phenotypic heterogeneity, metastasis, cooperation

Just as most groups, from the macro-structure of society down to a microbial community, rely on the presence of diverse individuals to function more effectively as a whole, tumor cells benefit from being genetically and phenotypically heterogenous. In cancer, genomic instability, the possibility of multiple stable gene network and epigenetic states, and differential access to resources depending on spatial location can all lead to the development of clonal subpopulations [1, 2]. Each subset of cells has different capabilities that together can allow a tumor to grow, recruit vasculature for the delivery of nutrients, evade the immune system, and spread to other parts of the body. While the dramatic reduction of cost in next generation sequencing has allowed for more access to genotyping of patient tumors and tumor subclones, the tools to investigate phenotypic heterogeneity are still being developed. Recently, there have been a number of interesting studies demonstrating new techniques to probe the question of cell behavioral divergence and cooperation, specifically in the study of metastasis.

The metastatic cascade is a complex, multi-step process that would require a cell to possess a varied set of skills if each cell had to pass all the hurdles on its own. The steps of metastatic progression can generally be divided into the primary tumor escape, involving the ability to migrate and invade through the tumor microenvironment, entering the nearby blood or lymphatic vasculature, resisting cell death while under shear in circulation, extravasation from circulation, including stopping and exiting at a secondary site, and finally survival and colonization at the new metastatic tumor location. While it is possible that one cell could acquire the necessary genetic mutations or epigenetic changes to exhibit every phenotype necessary to move through the entire metastatic process, another possibility is that successful metastasis relies on the cooperation of clonal subpopulations, using each subgroup’s primary behavior to benefit the whole tumor. Developing tools with the intent of sorting based on heterogeneity of cell behavior at each of these stages of metastasis instead of molecular biomarkers is improving our understanding of the metastatic cascade and how tumor cells may be working together to make it from their primary location to a distant metastatic site.

Migratory ability and metastatic ability are often mistakenly used interchangeably when discussing cancer cell behavior, but as we have noted above, a single cell’s migratory fitness may be important for primary tumor escape but have little to do with the likelihood of successful tumor metastasis. This point was demonstrated in our recent study where we used phenotypic sorting to produce stable subpopulations of weakly and highly migratory cells from an MDA-MB-231 parental cell line [3]. While the highly migratory cells injected into a mouse migrated locally from their primary tumor to a greater extent than the injected weakly migratory cells, the mice injected with weakly migratory cells underwent extensive metastasis to the lung, liver, and bone whereas minimal metastasis was observed in the mice that received the highly migratory cells. While the two subpopulations were both able to successfully complete the metastatic steps of primary tumor dissemination, survival in circulation, extravasation, and distant site colonization, the cells displaying the weakly migratory phenotype formed clusters in circulation and expressed high levels of E-Cadherin, both of which have been associated with worsened patient outcomes. Beyond studying divergence in cell’s migratory ability, other labs have also developed tools to fractionate cells phenotypically by their stiffness and their adhesive strength to investigate how different mechanical phenotypes affect a cell’s migratory and metastatic potential [4, 5]. Other groups have also used cutting edge imaging techniques to explore how epigenetic heterogeneity underscores phenotypic differences between leader and follower cells during collective cell migration [6]. By parsing out the differences in cell behavior, these groups are all contributing to our understanding of metastatic disease and how intratumoral heterogeneity contributes to a tumor’s ability to successfully metastasize.

Through our observation of clustering of weakly migratory cells leading to successful metastasis, as well as other work in the field studying leader-follower cell phenotypes and cancer fingers formed during collective cell migration, the importance of cooperation of phenotypically diverse cells is emerging [7]. While the possibility of phenotype switching and cell state plasticity could also play a role in cells’ ability to successfully metastasize, we should continue to study phenotypically different cells to learn how cancer cells take advantage of intratumoral heterogeneity to work together. Whether metastasis relies on the go-or-grow hypothesis, where cells are believed to switch between a migratory and proliferative phenotype during the different stages of metastasis, or it works more as a collective movement of go-ers and grow-ers, with migratory cells helping highly proliferative cells reach metastatic sites for colonization, or a combination of both, it is important that we continue to investigate phenotypic subpopulations, both separately and together.

## References

[1] Hanahan D, et al. Cell. 2011; 144:646–74. 10.1016/j.cell.2011.02.013. 21376230

[2] Brock A, et al. Nat Rev Genet. 2009; 10:336–42. 10.1038/nrg2556. 19337290

[3] Hapach LA, et al. Cancer Res. 2021; 81:3649–63. 10.1158/0008-5472.CAN-20-1799. 33975882 PMC9067366

[4] Stone NE, et al. Sci Rep. 2021; 11:18032. 10.1038/s41598-021-96862-y. 34504124 PMC8429413

[5] Beri P, et al. Cancer Res. 2020; 80:901–11. 10.1158/0008-5472.CAN-19-1794. 31857292 PMC7024658

[6] Summerbell ER, et al. Sci Adv. 2020; 6:eaaz6197. 10.1126/sciadv.aaz6197. 32832657 PMC7439406

[7] Zou H, et al. iScience. 2022; 25:103917. 10.1016/j.isci.2022.103917. 35252814 PMC8889141

